# The Conservation of VIT1-Dependent Iron Distribution in Seeds

**DOI:** 10.3389/fpls.2019.00907

**Published:** 2019-07-12

**Authors:** Seckin Eroglu, Nur Karaca, Katarina Vogel-Mikus, Anja Kavčič, Ertugrul Filiz, Bahattin Tanyolac

**Affiliations:** ^1^Department of Genetics and Bioengineering, Izmir University of Economics, Izmir, Turkey; ^2^Department of Bioengineering, Ege University, Izmir, Turkey; ^3^Department of Biology, University of Ljubljana, Ljubljana, Slovenia; ^4^Jozef Stefan Institute, Ljubljana, Slovenia; ^5^Department of Crop and Animal Production, Cilimli Vocational School, Duzce University, Duzce, Turkey

**Keywords:** biofortification, seed, iron, metal, vit1, plastid, synchrotron, homeostasis

## Abstract

One third of people suffer from anemia, with iron (Fe) deficiency being the most common reason. The human diet includes seeds of staple crops, which contain Fe that is poorly bioavailable. One reason for low bioavailability is that these seeds store Fe in cellular compartments that also contain antinutrients, such as phytate. Thus, several studies have focused on decreasing phytate concentrations. In theory, as an alternative approach, Fe reserves might be directed to cellular compartments that are free of phytate, such as plastids. However, it is not known if seed plastid can represent a major Fe storage compartment in nature. To discover distinct types of Fe storage in nature, we investigated metal localizations in the seeds of more than twenty species using histochemical or X-ray based techniques. Results showed that in Rosids, the largest clade of eudicots, Fe reserves were primarily confined to the embryo of the seeds. Furthermore, inside the embryos, Fe accumulated specifically in the endodermal cell layer, a well-known feature that is mediated by VACUOLAR IRON TRANSPORTER1 (VIT1) in model plant *Arabidopsis thaliana*. In rice, Fe enrichment is lost around the provasculature in the mutants of VIT1 orthologs. Finally, in *Carica papaya*, Fe accumulated in numerous organelles resembling plastids; however, these organelles accumulated reserve proteins but not ferritin, failing to prove to be plastids. By investigating Fe distribution in distinct plant lineages, this study failed to discover distinct Fe storage patterns that can be useful for biofortification. However, it revealed Fe enrichment is widely conserved in the endodermal cell layer in a VIT1-dependent manner in the plant kingdom.

## Introduction

One third of people suffer from anemia, with iron (Fe) deficiency being the most common reason (Kassebaum et al., 2014). Human diets are largely based on plants, which are often a poor source of Fe (Gibson et al., 2010). This is because corn, wheat, and rice kernels contain low concentrations of total Fe, and a large part of it cannot be absorbed by the human digestive system (Borg et al., 2009). To combat Fe deficiency, increasing bioavailable Fe (biofortification) of seeds has been suggested as the most sustainable approach (Shahzad et al., 2014).

Classical breeding, genetic engineering and other methods have been applied to increase Fe concentration in seeds (Murgia et al., 2012). Classical breeding has resulted in limited success, especially when natural cultivars show low variability in seed Fe levels. Genetic engineering methods have been shown to successfully increase Fe levels in major crops such as rice and wheat (Lee et al., 2009; Connorton et al., 2017). This increase has been mainly achieved by overexpressing proteins that boost the Fe storage capacity of the cells, eventually forcing the mother plant to divert more Fe into the seeds. Most of the biofortification approaches have targeted increasing concentration of Fe, but its low bioavailability is another major concern.

Bioavailability determines the portion of Fe that can be absorbed by the human digestive system. Fe absorption is affected by the form of Fe, which may lead to less than ten percent of the total Fe intake (Hurrell and Egli, 2010). Staple crop species accumulate Fe in cells that contain metal binding molecules, with phytate being the most important. Fe releases from phytate due to the presence of phytases to support germination (Hegeman and Grabau, 2001). However, humans do not possess phytase, therefore phytates act as antinutrients to humans. To increase Fe bioavailability, several studies have aimed at decreasing phytate concentrations (Shi et al., 2007; Reddy et al., 2017). Such a decrease has been achieved but at the expense of growth (Warkentin et al., 2012). An alternative approach could be to redirect Fe from phytate-containing compartments to phytate-free compartments. In nature, at a subcellular level, Fe accumulates in vacuoles, plastids, cytosol or elsewhere (Otegui et al., 2002; Cvitanich et al., 2010). In vacuoles, Fe accumulates in globules where it binds to phytate (Davila-Hicks et al., 2004; Lanquar et al., 2005). In plastids, Fe binds to ferritin protein (Waldo et al., 1995; Briat et al., 2010). In contrast to phytate-bound Fe, ferritin-bound Fe is bioavailable (Davila-Hicks et al., 2004). Thus, optimizing seed Fe for human nutrition requires not only increasing Fe concentration but also its bioavailability, which is strictly dependent on its subcellular level distribution.

The advances in Fe imaging techniques have allowed detailed investigations of Fe localization in the small seeds of *Arabidopsis thaliana* (Eroglu, 2018). The X-ray synchrotron fluorescence imaging technique identified nonhomogenous distribution of Fe throughout the embryo and revealed an Fe-enriched hotspot around the provascular strands (Kim et al., 2006). However, the emerging technique failed to pinpoint the specific cells that are overaccumulating Fe. Such a resolution has been achieved with the use of an enhanced version of classical Perls staining, namely, Perls/DAB (Roschzttardtz et al., 2009). Perls/DAB revealed that the Fe enrichment around provascular strands is confined to endodermal cells (Roschzttardtz et al., 2009; Ramos et al., 2013).

The genetic basis of endodermal Fe accumulation has been tackled by the comparison of T-DNA insertion mutants of *A. thaliana*. Two individual lines lost the endodermal Fe enrichment when mutated in the *VACUOLAR IRON TRANSPORTER1 (VIT1)* gene (Kim et al., 2006; Eroglu et al., 2017). *VIT1* localized to the tonoplast, expressed in the stele, and complemented the Fe hypersensitivity of yeast when expressed heterologously; suggesting it imports Fe into the vacuoles of endodermal cells (Kim et al., 2006). Disruption of VIT1 relocalized Fe from the endodermal to the subepidermal cell layer in *vit1* mutants. *vit1* mutants further lost the subepidermal Fe enrichment when mutated in *METAL TOLERANCE PROTEIN8 (MTP8)* (Eroglu et al., 2016, 2017; Eroglu, 2018). Similar to *VIT1*, *MTP8* localized to the tonoplast and complemented the Fe hypersensitivity of yeast (Eroglu et al., 2017). The disruption of *VIT1* and *MTP8* together relocalized Fe throughout the embryo homogeneously. In conclusion, analysis of loss-of-function mutants of *A. thaliana* shows that metal transporters determine where metals will be stored in the mature seed. This indicates that if a suitable Fe transporter protein is used, it might be possible to direct Fe to certain organelles that are free of antinutrients.

Our research aim was to identify a seed Fe transporter that can be useful to direct Fe to the plastids. *A. thaliana* accumulates Fe almost exclusively inside the vacuoles, therefore failing to be a suitable model for such an approach. Fe distribution in the seeds of other plants is poorly known. To find plastid targeting Fe transporters, we screened Fe distributions in seeds belonging to distinct plant lineages. Based on what is known from the model plant, we hypothesized that Fe concentrated regions will indicate the presence of a transporter. To this end, we examined the seeds of more than 20 different species belonging to several different plant orders by using Perls/DAB histochemical staining or synchrotron X-ray fluorescence spectroscopy.

## Materials and Methods

### Collection of Seeds

Seeds were obtained from gene banks, local suppliers, or personally collected (Table 1). Rice T−DNA insertion mutants *vit1-1* (*Os04g0463400* Zhonghua11 background) and *vit2-1* (Knock out line of Os09g0396900, Dongjin genetic background) and wild type (Dongjin) were courtesy of Dr. Ji-Ming Gong (Zhang et al., 2012).

**TABLE 1:** Seeds used in the study.

**Species**	**Source**
*Linum usitatiss*	National Genebank, Menemen, Turkey
*Medicago truncatula*	National Genebank, Menemen, Turkey
*Cucumis sativus*	National Genebank, Menemen, Turkey
*Citrullus lanatus*	National Genebank, Menemen, Turkey
*Cannabis sativa*	National Genebank, Menemen, Turkey
*Euonymus europaeus*	National Genebank, Menemen, Turkey
*Alyssum sibiricum*	National Genebank, Menemen, Turkey
*Brassica oleracea*	National Genebank, Menemen, Turkey
*Gossypium arboreum*	National Genebank, Menemen, Turkey
*Batis maritima*	Kew Gardens, London, England
*Moringa peregrina*	Kew Gardens, London, England
*Eucalyptus elata*	Kew Gardens, London, England
*Geranium carolinianum*	Kew Gardens, London, England
*Brassica napus*	Genebank, Gatersleben, Germany
*Limnanthes douglasii*	Botanischer Garten, Marburg, Germany
*Arabidopsis thaliana*	Arabidopsis Biological Resource Center, Ohio, United States
*Reseda lutea*	Personally collected^*^
*Noccaea praecox*	Personally collected^∗∗^
*Carica papaya*	Commercial
*Arachis hypogaea*	Commercial
*Solanum lycopersicum*	Commercial
*Capparis spinosa*	Commercial

^*^Çilden et al., 2018^∗∗^Collected from Pb, Zn polluted site, Mežica, Slovenia (Vogel-Mikus et al., 2005).

### Perls Staining and DAB/H_2_O_2_ Intensification (Perls/DAB)

Wherever possible (embryos were large, could easily be isolated, seed coat was sufficiently soft to be cut, and high magnification was not needed), samples were directly (i.e., without fixation) stained by Perls (Figures 4A,B) or Perls/DAB (Figures 2C, 3D) (Roschzttardtz et al., 2009). In other cases, *in situ* protocol was used. For direct Perls staining, samples were vacuum infiltrated in 4% (v/v) HCl and 4% (w/v) K-ferrocyanide (Perls stain solution) for 15 min and incubated for 30 min at room temperature (Stacey et al., 2008). If the Fe signal was weak, it was intensified using DAB (Roschzttardtz et al., 2009). Perls-stained samples were incubated in a methanol solution containing 0.01 M NaN_3_ and 0.3% (v/v) H_2_O_2_ for 1 h, and then washed with 0.1 M phosphate buffer (pH 7.4). For the intensification reaction, samples were incubated between 10 and 30 min in a 0.1 M phosphate buffer (pH 7.4) solution containing 0.025% (w/v) DAB, 0.005% (v/v) H_2_O_2_, and 0.005% (w/v) CoCl_2_. The reaction was stopped by rinsing with distilled water.

For *in situ* Perls staining, seeds were first fixed in 10% formalin and then dehydrated in an ethanol series (30%, 40%,…, 100%). Seeds were cleared with xylene. Seeds that were still too hard to cut were further incubated in 20% formic acid for 45 min. Seeds were then embedded in wax and 10–30 μm sections were cut. Sections were deparaffinized by incubation at 65^∘^C for 15 min following a xylene treatment for 15 min, and were stained with Perls/DAB according to Roschzttardtz et al. (2009). Sections were treated with 4% (v/v) HCl and 4% (w/v) K-ferrocyanide (Perls stain solution) for 15 min and incubated for 30 min at room temperature (Stacey et al., 2008). DAB intensification was applied as described in Meguro et al. (2007). For the intensification reaction, the sections were incubated between 10 and 30 min in a 0.1 M phosphate buffer (pH 7.4) solution containing 0.025% (w/v) DAB (Sigma), 0.005% (v/v) H_2_O_2_, and 0.005% (w/v) CoCl_2_ (intensification solution). The reaction was stopped by rinsing samples with distilled water. Samples were imaged with a light microscope (Axioskop; Carl Zeiss, Jena, Germany).

For iodine staining, sections were stained with Gram’s iodine solution (6.7 g potassium iodide and 3.3 g iodine dissolved in 1L H2O; Ward’s science) for at least 5 s.

For Naphthol Blue–Black staining of proteins, slides were placed in 0.1% (w/v) Naphthol Blue–Black, 10% (v/v) acetic acid for 5 min. For immunohistochemistry, after deparaffinization, tissues were rehydrated by ethanol series and hybridization was conducted according to Paciorek et al. (2006). Anti-rabbit secondary antibody was conjugated with Alexa-488 and was used with 1:800 dilution (Jackson ImmunoResearch, West Grow, PA, United States).

### X-Ray Fluorescence

X-ray fluorescence imaging of *Brassica napus*, *Moringa peregrine*, and *Euonymus europaeus* was performed at XRF beamline of Synchrotron Elettra (Karydas et al., 2018) on 60 μm seed cross-sections. The seeds were imbibed overnight at 4°C, flash frozen in propane cooled with liquid nitrogen, embedded in tissue freezing medium (Leica, Germany), and cut using CM3060 Leica Cryostat (Vogel-Mikuš et al., 2014). The sections were sandwiched between two 2.5 μm Mylar foil and scanned by 75 × 75 μm beam at an excitation energy of 10 KeV. Obtained XRF spectra were fitted by PyMCA software (Solé et al., 2007) and quantified (Kump and Vogel-Mikuš, 2018). Imaging of element distribution in *Noccaea praecox* was performed by micro-PIXE as described (Vogel-Mikus et al., 2007; Vogel-Mikuš et al., 2008).

### Bioinformatics Analyses

VIT1 protein sequences were derived from the Phytozome database 12.1.6 version^1^ (Goodstein et al., 2011) for bioinformatics analyses. The following plants were included: *A. thaliana* (AT2G01770), *Carica papaya* (evm.model.supercontig_2.168), *Cucumis sativus* (Cucsa.174130.1, Cucsa.012090, and Cucsa.012100), *Linum usitatiss* (Lus10021809), *Medicago truncatula* (Medtr8g105790 and Medtr8g105810), *Eucalyptus grandis* (Eucgr.B02690, Eucgr.B02691, and Eucgr.G02084), *B. napus* (A0A078JDR3), *Gossypium arboreum* (A0A0B0MD68), *Arachis duranensis* (XP_020996537.1), *Arachis ipaensis* (XP_020977382.1), *Brachypodium distachyon* (Bradi4g29720 and Bradi5g12570), *Brassica rapa* (Brara.B03068 and Brara.F03456), *Chlamydomonas reinhardtii* (Cre02.g099500 and Cre02.g107550), *Glycine max* (Glyma.05G24060 and Glyma.08G047500), *Gossypium raimondii* (Gorai.013G029700), *Zea mays* (GRMZM2G074672 and GRMZM2G107306), *Vitis vinifera* (GSVIVG01011628001 and GSVIVG01011629001), *Oryza sativa* (LOC_Os04g38940 and LOC_Os09g23300), *Phaseolus vulgaris* (Phvul.002G322800 and Phvul.002G322900), *Populus trichocarpa* (Potri.010G104100 and Potri.010G104200), *Physcomitrella patens* (Pp3c2_34540), *Prunus persica* (Prupe.1G335200 and Prupe.1G335300), *Sorghum bicolor* (Sobic.002G194600 and Sobic.006G109000), and *Solanum lycopersicum* (Solyc04g008060). Protein domains were detected using Pfam 31.0^2^ (Finn et al., 2015). Sequence length, molecular weight, and isoelectric point (*pI*) were determined by using the ExPASy ProtParam tool^3^ (Gasteiger et al., 2005). The transmembrane helices were predicted by using The HMMTOP transmembrane topology prediction server version 2.0^4^ (Tusnady and Simon, 2001). The phylogenetic tree of VIT1s was constructed with MEGA 7.0.2 software (Kumar et al., 2016) using maximum likelihood (ML) method with 1000 bootstraps. The evolutionary distances were computed using the Poisson correction method (Zuckerkandl and Pauling, 1965). Identity values (%) of VIT1s were analyzed using the NCBI blastp tool.^5^

*In silico* expression data of soybean *VIT1* were obtained using the Seed Gene Network Database (www.seedgenenetwork.com). Arabidopsis *VIT1* gene number AT2G01770 was submitted. That submission returned soybean (*G. max*) GeneChip Expression Profile for Probe Set – Gma.6465.1.S1_a_at corresponding to *Glyma01g40600*. The signal data of individual experiments were obtained from the original file (Geo number: GSE46906).

## Results

### Fe Accumulates in Endodermis in Brassicaceae Family

**FIGURE 1:**
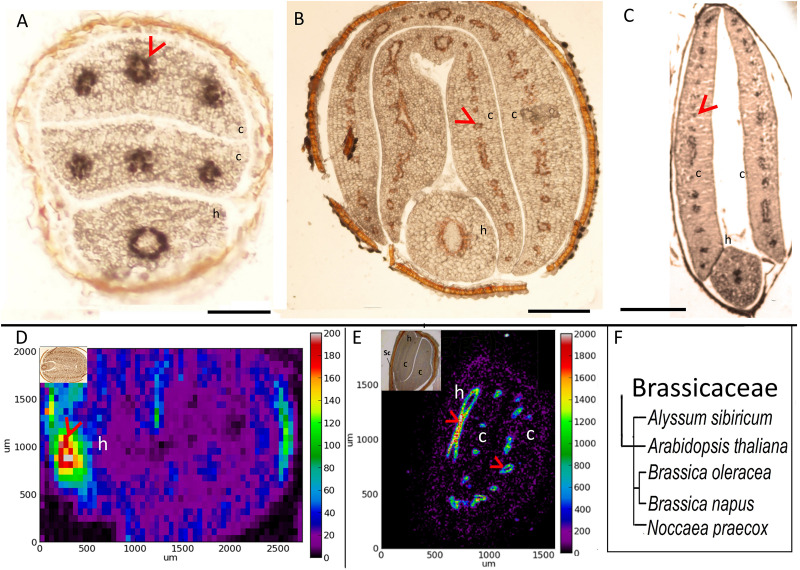
Fe accumulates in the endodermis of Brassicaceae family. **(A–C)** Perls/DAB-stained seed cross sections. Cross sections were stained with Perls/DAB and observed under light microscope. From left to right: *Arabidopsis thaliana*, *Brassica oleracea*, and *Alyssum sibiricum*. Brown regions surrounding the embryos are seed coats. These were already brown before the staining; thus, the color does not reflect the stained Fe. Fe appeared as black stains in panels **(A)** and **(C)**, and brown in panel **(B)**. Circular-shaped staining in *Arabidopsis thaliana* corresponds to the endodermal cells surrounding the provascular strands. **(D,E)** Synchrotron X-Ray fluorescence images of relative Fe distribution in the seeds. **(D)** *Brassica napus* and **(E)** *Noccaea praecox.* **(F)** Branch of the taxonomic tree, for the whole tree refer to Figure 8. This branch shows species that were examined for Fe reserves in Brassicaceae family and used as a visual aid. Note that *Arabidopsis thaliana*, *Alyssum sibiricum*, and *Noccaea praecox*, consist of a single pair of cotyledons, but both *Brassica napus* and *Brassica oleracea* consist of a pair of inner and an outer cotyledons. Bar represents 0.1 mm in panel **(A)**, 0.5 mm in panels **(B,C)**. c, cotyledon; h, hypocotyl. Red arrow heads point to examples of specific Fe accumulation pattern, closed rings around provasculature of cotyledons **(A–C,E),** and hypocotyl **(D)**.

To investigate how Fe distribution differs between *A. thaliana* and other members of Brassicaceae, by using Perls/DAB method and X-ray synchrotron analysis, we compared Fe distribution in several different Brassicaceae species. *A. thaliana* showed circular Fe-enriched regions in cotyledons and hypocotyl (Figure 1A), confirming previous reports (Kim et al., 2006; Eroglu, 2018). This region corresponds to the endodermis (Roschzttardtz et al., 2009). Similar to *A. thaliana* (Figure 1A), all other members of Brassicaceae showed circular Fe-enriched regions, both in cotyledons and the hypocotyl (Figures 1B–E). Fe rings seemingly overlap the region surrounding provascular bundles, most probably endodermis, based on extrapolation from *A. thaliana*. *B. napus*, and *Brassica oleracea* contained conduplicate cotyledons (i.e., inner and outer), both of which exhibited the same Fe distribution pattern (Figures 1B,D). Despite the similarities in these patterns, variations were also observed between species. In contrast to *A. thaliana* (Figure 1A), the Fe-enriched region of *B. napus* was not confined to a single cell layer in the hypocotyl (Figure 1C). Furthermore, *Alyssum sibiricum* showed two adjacent Fe-enriched circles instead of one in its hypocotyl (Figure 1D). Taken together, results showed that not only *A. thaliana*, but also other Brassicaceae species store main Fe reserves in the endodermis.

### Conservation of Fe-Enriched Endodermis in the Order Brassicales

**FIGURE 2:**
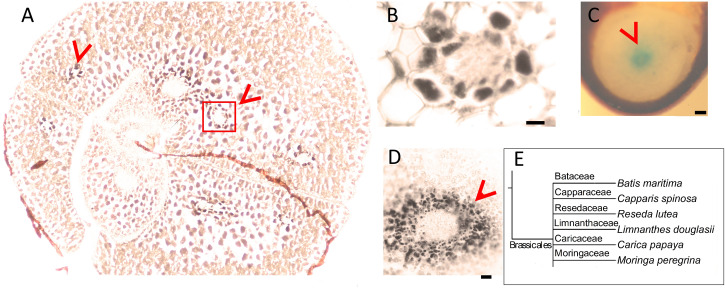
Fe-enriched endodermis is conserved in Brassicales order. Seed cross sections were stained either with Perls or with Perls/DAB and observed under light microscope. **(A)** *Limnanthes douglasii*. **(B)** Higher magnification of the red square in panel **(A)**. **(C)** Hypocotyl of *Capparis spinosa*. **(D)** Hypocotyl of *Batis maritima*. **(B–D)** Focusses on vascular tissues. Fe staining appeared black in panels **(A,B,D)** and blue in panel **(C)**. Brown colored structure partially surrounding the embryo in panel **(A)** is the seed coat. This was already brown before the staining; thus, the color does not reflect the stained Fe. This branch shows species that were examined for Fe reserves in Brassicaceae family and used as a visual aid. Bar is 0.1 mm for panel **(A,C)**; 0.01 mm for panel **(B,D)**. **(E)** Branch of the taxonomic tree, for the whole tree refer to Figure 8. This branch shows species that were examined for Fe reserves in the same order but different families and used as a visual aid. Red arrow heads point to examples of specific Fe accumulation pattern, closed rings around provasculature of cotyledons **(A,B)** and hypocotyl **(C,D)**.

Next, we assessed whether the conserved Fe pattern in *A. thaliana* seed extends beyond the family level to the order level. We searched for ring-like Fe distribution patterns around the provasculature of either the cotyledon or the hypocotyl, which was typical of Brassicaceae. We stained distinct species belonging to Brassicales by either Perls alone (for Fe-rich samples) or with DAB intensification (for Fe-poor samples) to reach a balance in staining intensity. In *Limnanthes douglasii* embryos, all cells showed the staining. At first glance, stained cells did not exclusively correspond to the endodermis (Figure 2A). However, closer examination revealed that cells around the provascular bundles of cotyledons were slightly enriched with Fe (Figure 2B). In *Capparis spinosa*, Perls staining without DAB amplification revealed that Fe accumulated close to the central cylinder of the hypocotyl (Figure 2C). In *Batis maritima*, Fe accumulated in several cell layers surrounding the provasculature, including the endodermis (Figure 2E). Taken together, an Fe-enriched endodermis is conserved in plants at least in order level.

### Fe Accumulation in the Endodermis Beyond the Order Brassicales

**FIGURE 3:**
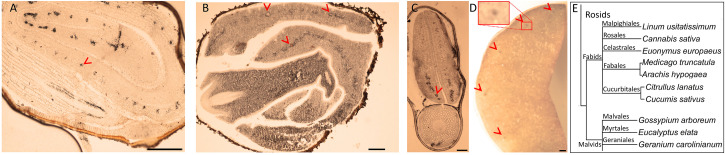
Fe-enriched endodermis is conserved in distinct plant orders. Seed cross sections belonging to members of various orders were stained with Perls/DAB and observed under light microscope. From panels **(A–C)**, *Gossypium arboreum*. *Eucalyptus elata* and *M. truncatula*, respectively. **(D)** Single cotyledon of *Arachis hypogaea*. Fe staining appeared black in panels **(A–C)** and brown in panel **(D)**. Outer brown–black cover in panels **(A–C)** are seed coats. These were already brown before the staining, thus the color do not reflect the stained Fe. In panel **(A)**, longitudinal section of hypocotyl and cross section of folded cotyledons can be differentiated. Red arrow heads show Fe enrichment surrounding the provascular strands of cotyledons. **(E)** Branch of the taxonomic tree, for the whole tree refer to Figure 8. This branch shows species that were examined for Fe reserves in distinct orders and used as a visual aid. Note that only selected examples from panel **(E)** is shown through **(A–D)**. Bar is 1 mm for panel **(A)** and 0.1 mm for panels **(B–D)**. Red arrow heads point to examples of specific Fe accumulation pattern, closed rings around provasculature of cotyledons.

Brassicales with 16 other orders together constitute a large clade of flowering plants, namely, Rosids (Chase et al., 2016). We next pursued the Fe distribution in species that belong to Rosids (Table 1). *G. arboreum* showed ring-like Fe distribution around provasculature (Figure 3A), similar to typical Fe-stained endodermal cells. Furthermore, the rest of the cells were devoid of staining, indicating endodermis represented the main Fe reserves in the embryo. Likewise, *Eucalyptus elata* also showed Fe enrichment in the endodermis of the cotyledon. However, that of hypocotyl did not show the same enrichment (Figure 3B). We examined two species that belong to the Fagales order, namely, *M. truncatula* and *Arachis hypogaea* (Figures 3C,D). *M. truncatula* showed circular Fe-stained regions in the cotyledons, which represented the largest Fe pool in the embryo (Figure 3C). In contrast to *M. truncatula*, Fe seemingly accumulated homogeneously in *A. hypogaea* However, closer examination of *A. hypogaea* revealed provascular strands were enriched in Fe (Figure 3D).

### Conservation of VIT1 Sequence in Species That Do Not Show an Fe-Enriched Endodermis

**FIGURE 4:**
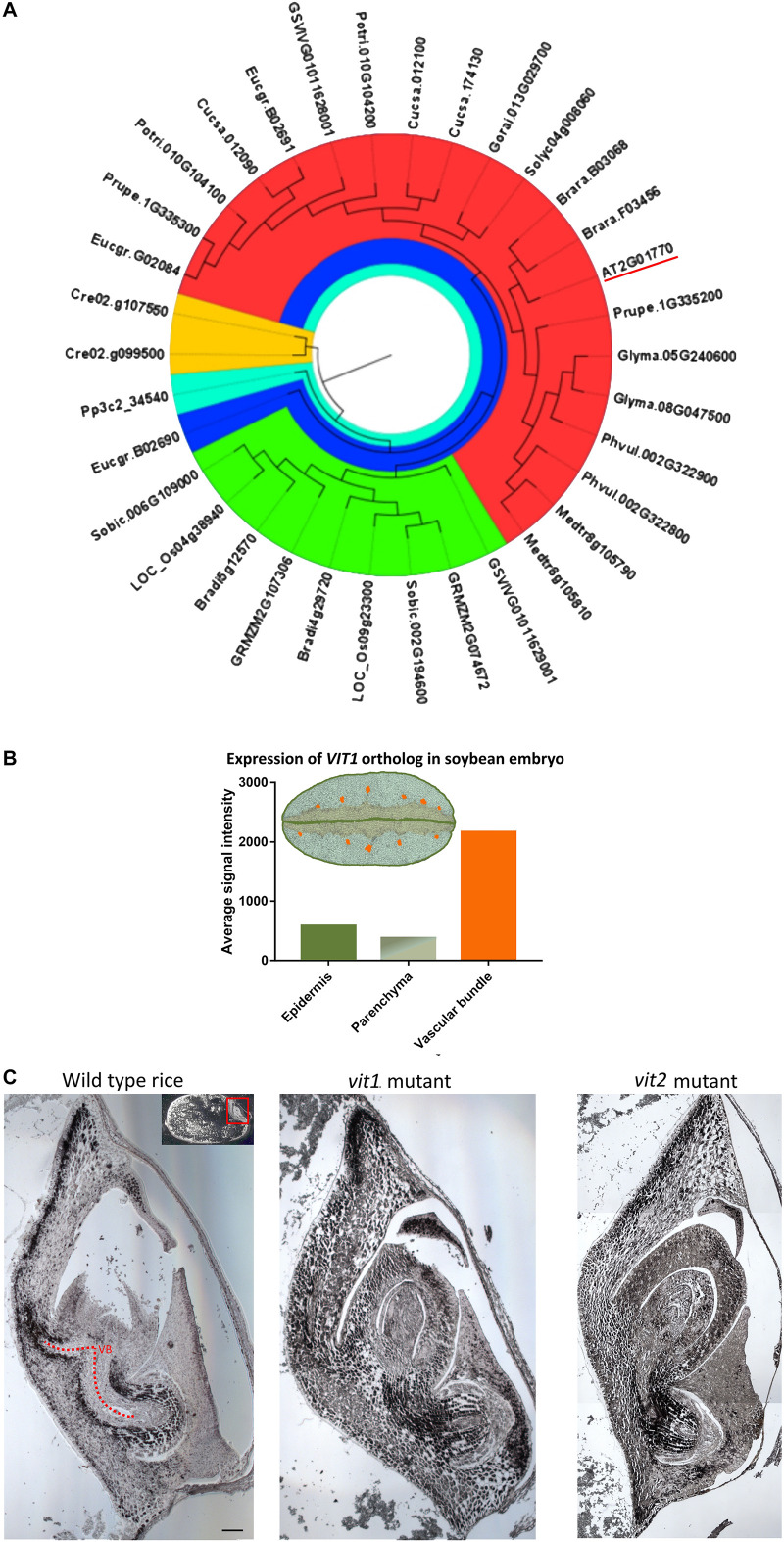
Conservation of VIT1 sequence, expression and function. **(A)** Phylogenetic distribution of VIT1 orthologs in 18 different plant species from monocots, dicots and lower plants. Phylogeny was constructed by MEGA 7 with ML method for 1000 bootstraps using putative 34 VIT1 protein sequences. The red segment includes only dicots. Green segment includes only monocots except for one member of *Vitis vinifera*. The blue segment shows a diverged dicot member of *E. grandis*. Orange and cyan colors show algal and moss species, respectively. VIT1 from *Arabidopsis thaliana* is underlined. For details on analyzed sequences, refer to materials and method section. **(B)** Expression of *VIT1* ortholog (*Glyma01g40600*) in embryo tissues of soybean (Microarray data retrieved from www.seedgenenetwork.net). The picture illustrates the soybean (*Glycine max*) tissues: green, epidermis; orange vascular bundles; rest, parenchyma. **(C)** Impact of *VIT1* orthologs in Fe distribution of rice (*Oryza sativa*) embryo. Unlike dicots, monocots such as rice do not have well defined organs. Longitudinal sections of whole rice embryos were obtained from either wild type or mutant rice (*VIT1* or *VIT2* genes were either substantially knocked down or knocked out), stained by Perls/DAB (black color) and Fe distribution in the embryos were compared. Region with red line corresponds to provascular bundles (VB) in rice embryo. Red arrow heads point specific Fe accumulation pattern around provasculature. Bar is 0.1 mm.

In *A. thaliana* seed, Fe enrichment in the endodermis is dependent on a functional VIT1 protein (Kim et al., 2006). We failed to observe an Fe-enriched endodermis in some species. To evaluate how conserved VIT1 is in these species, VIT1 protein sequences in distinct plants were compared (Figure 4). First, to get a general picture, 34 sequences were determined and compared from 18 plant species (Figure 4A). In contrast to *A. thaliana*, which had only one VIT1, most plants showed two copies. Interestingly, in *V. vinifera*, one of the two copies of VIT1 clustered with monocot VITs in the phylogenetic tree. *E. grandis* contained three homologs of VIT1, one of which was distinctly diverged. These data suggest that VIT1 is well-conserved in distinct plant lineages (Supplementary Figures 1, 2).

We next assessed the conservation of VIT1 protein sequence in species that were used in Fe staining. The whole genome sequence was available only for *A. thaliana*, *C. papaya*, *Cucumis sativus*, *L. usitatiss*, *M. truncatula*, *E. grandis*, *B. napus*, *G. arboreum*, and *Arachis sp.* (Supplementary Figure 3). VIT1 sequences of all ten species consisted of 245–265 amino acids with five transmembrane helices and clustered in three subgroups (Supplementary Table 1). All contained the VIT1 domain structure (PF01988) (Supplementary Figure 2). Protein BLAST analyses showed that identity values (%) of *Arabidopsis* and the other nine VIT1s ranged from 29 to 92% (Supplementary Figure 3). We found the highest identity value between *Arabidopsis* and *B. napus* (92%), followed by *M. truncatula* and *Linum usitasiss* (83%), while the lowest was found between *A. thaliana* and *Arachis* spp. (29%), indicating variations of *VIT1* genes in plants. All three VIT1 subgroups included members that stored Fe around the provasculature. Therefore, although some species did not exhibit a VIT1-dependent Fe enrichment in the endodermis (Supplementary Figure 4), their VIT1 sequences did not diverge from others.

In *A. thaliana*, analysis of *ProVIT1::GUS* lines showed *VIT1* is preferentially expressed in the provasculature (Kim et al., 2006; Eroglu et al., 2017). To investigate whether spatial VIT1 expression is conserved in VIT1 orthologs, we compared expression of *VIT1* orthologs using publicly available microarray databases. We found that *VIT1* signal was highest in the provasculature in soybean embryo (Figure 4B). This data indicated spatial expression pattern is conserved in VIT1 orthologs.

Furthermore, to investigate whether VIT1’s function is also conserved, we compared Fe distributions in mutants of VIT1 orthologs. VIT1 orthologs have been reported to involve in Fe distribution in rice embryo (Zhang et al., 2012; Bashir et al., 2013); however, precise description of the effect was lacking due to the low resolution, where Perls staining was applied without DAB amplification. Perls/DAB staining revealed that Fe specifically concentrated around the provasculature in wild type rice and not in single *vit1* nor *vit2* mutants (Figure 4C). Fe accumulated less heterogeneously among embryonic tissues in those compared to the wild type. This data suggested Fe enrichment around provasculature is conserved and mediated by VIT1 homologs in rice.

### Organ, Tissue, and Subcellular Level Variations in Seed Fe Storage

**FIGURE 5:**
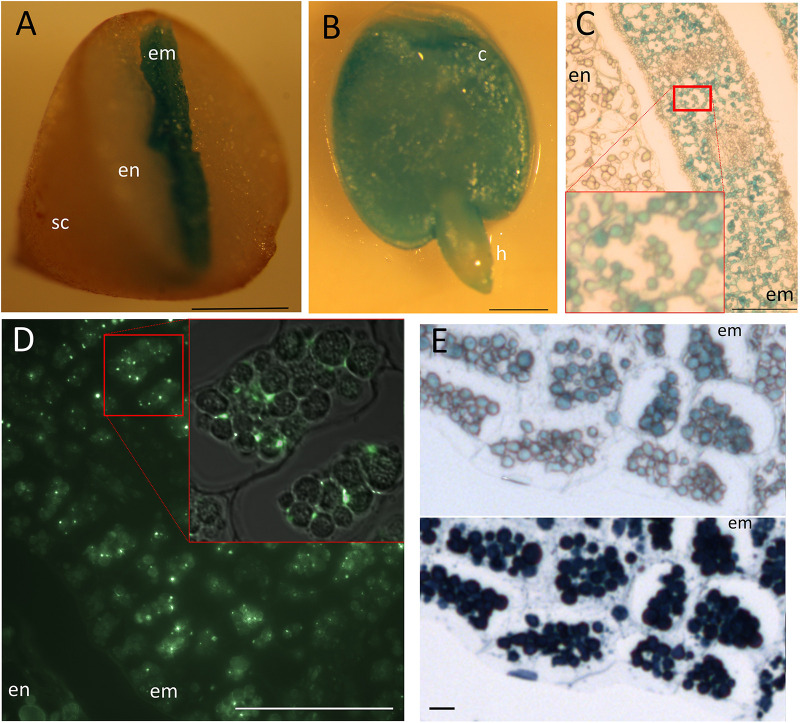
Fe storage in *Carica papaya*. Distribution of Fe as subcellular hotspots in *Carica papaya* seeds. *Carica papaya* seeds were cleaned from the pericarp. **(A)** Seeds were cut into half by hand and were stained by Perls. **(B)** Whole embryo was isolated from the seed and was stained by perls. **(C)** Perls-stained thin cross section of a cotyledon. Red squares indicate close up. **(D)** Immunohistochemistry using ferritin antibodies. Bright green spots indicate ferritin presence. Red square indicates closeup, fluorescence image overlayed on top of the light microscopy image to indicate localization of fluorescent spots with regards to the Fe storing organelles. **(E)** Juxtaposition of Fe-stained and protein-stained regions of the embryo cross sections. The same cross section was first stained by Perls, pictured (top panel) and then stained by Naphthol Blue–Black, pictured again (bottom panel). Blue color in panel **(A–C)** and in the top panel of **(E)** indicate stained Fe. Note, the cellular position and number of the organelles slightly differ in panel **(C)**, compared with **(D)** and **(E)**. The difference might be due to a fixation artifact or cross sectioning from distinct regions of the embryos. Dark blue color in the bottom panel of **(E)** indicates presence of proteins. sc, seed coat; en, endosperm; c, cotyledon; h, hypocotyl; em, embryo. Bar is 1 mm for panels **(A,B)**; 0.05 mm for panel **(D)**; 0.1 mm for panel **(C)**; 0.01 mm for panel **(E)**.

Perls staining revealed Fe storing protein bodies show great variation in the structure. *C. papaya* seed showed intense staining in the center corresponding to the embryo – even though the DAB intensification step was skipped –; while the peripheries were devoid of staining, corresponding to the endosperm (Figures 5A,B). At the tissue and subcellular level, staining was confined to organelles similar to leucoplasts regarding the shape, size, and number (Figure 5C). Distribution of this organelle did not differ between the endodermis and other cell layers. Since a specific group of seed leucoplasts store starch (i.e., amyloplast), we next investigated whether Fe-accumulating organelles belong to that group. Iodine and periodic acid could only weakly stained those (data not shown), failing to show these organelles accumulate starch. Next, since Fe in plastids is associated with ferritin, using immunohistochemistry, we investigated if Fe accumulating organelle contains ferritin. Ferritin fluorescence were observed in the embryo cells but outside of those organelles (Figure 5D). Instead, unlike plastids, these organelles accumulated proteins, as shown by Naphthol Blue–Black staining (Figure 5E). Taken together, these data suggested accumulating organelles in *C. papaya* were indeed protein storage vacuoles or globules similar to many other plant seeds, despite their distinct relative size and appearance.

**FIGURE 6:**
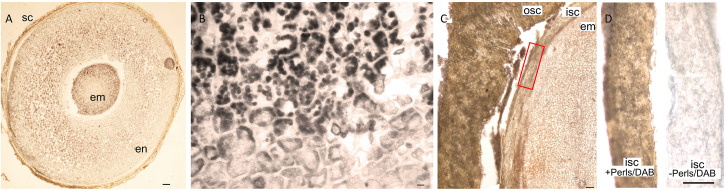
Unusual Fe storage in the endosperm and in the seed coat. Fe staining in *Euonymus europaeus* and *Moringa peregrina.* Cross sections were stained with Perls/DAB and observed under light microscope. **(A)** *Euonymus europaeus*. **(B)** A close-up to endosperm tissue of *Euonymus europaeus*. **(C)** *Moringa peregrina*, focused on a small part of the seed. Embryo, inner and outer sections of the seed coats are visible. *Moringa peregrina* does not possess an endosperm and has a thick two-layered seed coat. **(D)** Dissected inner seed coats of *Moringa peregrina.* Left, Perls/DAB stained, right unstained control. Fe staining appeared brown in panels **(A,C,D)** and black in panel **(B)**. Seed coat in panel **(A)** and outer seed coat in panel **(C)** were already brown before the staining, thus the color does not reflect the stained Fe. In contrast, inner seed coat of *Moringa peregrina* is white, and brown color in panel **(C)** shows the stained Fe. sc, seed coat; en, endosperm; osc, outer seed coat; isc, inner seed coat; em, embryo. Bar is 0.1 mm for panel **(A)**, 0.01 mm for panel **(B)**, 1mm for panels **(C,D)**.

Nutrients accumulate usually in embryo part of the seeds. We found that in addition to the embryo, the endosperm and even the seed coat can significantly store Fe in a few Rosid species. For example, *E. europaeus* contained a large endosperm, which was intensely stained by Perls/DAB (Figures 6A,B). As another example, the seed coat of *Moringa peregrina* was stained by Perls/DAB (Figures 6C,D). Interestingly, the staining intensity was higher in its seed coat compared with its embryo (Figure 6C), indicating that the seed coat is a major Fe accumulation site in *M. peregrina*.

**FIGURE 7:**
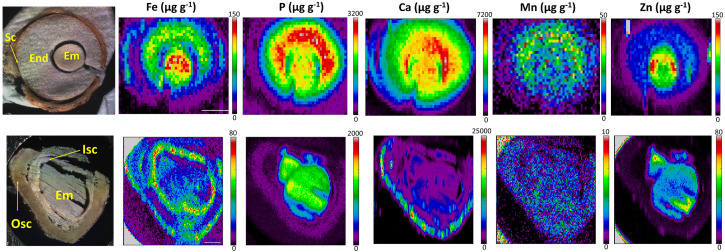
X-ray fluorescence spectroscopy confirms endosperm and seed coat accumulate Fe. Spatial distribution of metals in *Euonymus europaeus* (**top panel**) and *Moringa peregrina* (**bottom panel**). The first column of images shows light microscopy images. Other columns show distribution of elements; Fe, P, Ca, Mn, and Zn, respectively. Colorbar indicates concentration. sc, seed coat; en, endosperm; isc, inner seed coat; osc, outer seed coat; em, embryo. Bar is 1 mm.

Finally, the distinct distribution of Fe in *E. europaeus* and *M. peregrina* seeds was further investigated by synchrotron X-ray fluorescence spectrometry. Synchrotron X-ray fluorescence confirmed the Fe accumulation in the endosperm of *E. europaeus* and in the inner seed coat of *M. peregrina* (Figure 7). In addition to Fe, synchrotron analysis further revealed the distribution of other metals. In *E. europaeus*, phosphorus (P) and Mn localized homogeneously through the embryo and endosperm, while zinc (Zn) localized exclusively in the embryo. In *M. peregrina*, Fe concentration was high not only in the inner seed coat as previously revealed by Perls/DAB (Figure 6) but also in the outer seed coat (Figure 7). Fe localized specifically to the outer side of the outer seed coat and throughout the inner seed coat. The outer seed coat of *M. peregrina* accumulated calcium (Ca), spatially overlapping Fe, but not other metals. Zn accumulated exclusively in the embryo.

## Discussion

We screened seeds that belong to distinct plant lineages using Perls/DAB staining and X-ray-based methods to find Fe-accumulating plastids. Although we failed to identify such seeds, our approach revealed seed Fe storage patterns in distinct plant lineages. Among those, Fe enrichment around provasculature is well conserved in Rosids which is dependent on orthologs of VIT1.

**FIGURE 8:**
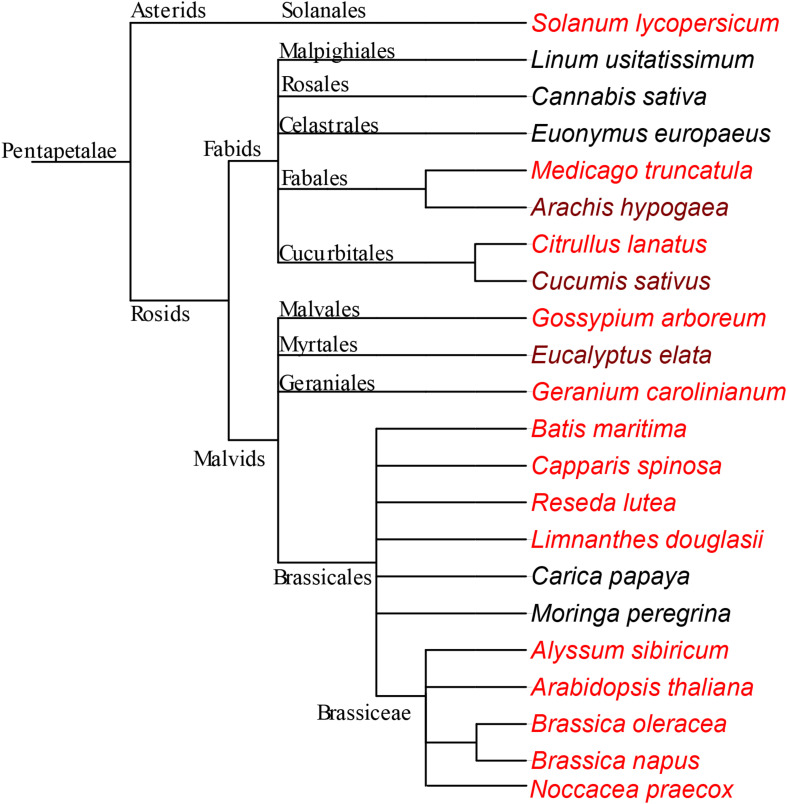
Fe-enriched endodermis is a conserved feature in Rosids. Schema representing species that are used in this study. The phylogenetic tree was generated using NCBI taxonomy database and exported with the version 3 of the Interactive Tree of Life (iTOL) (Letunic and Bork, 2016). Both dark and light red colored species were enriched with Fe around provascular strands. In the darker-red-colored species, endodermis Fe does not represent the highest concentration of Fe, in contrast to the lighter-red-colored species. The species which are not red, do not specifically accumulate large Fe reserves in their endodermis. See discussion for the interpretation of the negative data.

Fe accumulates almost exclusively in the embryos in Rosids (Figures 1–3, 5 and in all other plants that were examined in Figure 8). In contrast, monocots store large quantities of Fe also in the endosperm, specifically in the aleurone layer (Lu et al., 2013; Singh et al., 2013, 2014; Vatansever et al., 2017). This organ level difference in Fe partition may be explained by the decrease in endosperm size during evolution (Forbis et al., 2002; Finch-Savage and Leubner-Metzger, 2006). Branches that appear early, such as monocots, almost never possess an embryo occupying more than half of the total seed volume. In contrast, branches that appeared later (e.g., Rosids) can possess embryo that may fill almost the entire seed. In the latter, the endosperm shrinks as the seed develops and the embryo eventually occupies most of the seed volume. Since the endosperm is a nutritive tissue, as it degrades, nutrient storage function must be taken over by the embryo itself. Therefore, we suggest that the metal accumulation in the endosperm, at least for Fe, is taken over by the embryo as an evolutionary trend.

Besides the general trends, Fe may accumulate in distinct organs and cellular compartments. The destination of nutrients must be the embryo, as the mature seed coat is dead while the endosperm is sterile. However, Fe accumulates in the seed coat higher than the embryo in rare cases (Figures 6, 7; Cvitanich et al., 2010; Moraghan et al., 2002). This can be related to the presence of chelators in the seed coat, such as tannins, which immobilize metal nutrients (Lombardi-Boccia et al., 1995). The function of trapped Fe reserves in the seed coat should be addressed in the future. In this regard, since most seed coats are naturally colored; Perls staining may fail to detect Fe; however, this problem can be circumvented by using fluorescent dyes (Park et al., 2014). In contrast to Fe, Zn is not trapped in the seed coat (Figure 7). This might be due to its lower affinity to tannins and other polyphenols (Santos-Buelga and Scalbert, 2000). In Rosids, the endosperm rarely stores a significant amount of Fe (only in a few species, see Figures 6A,B, 7 and Supplementary Figure 4). Interestingly, X-ray fluorescence showed Fe in the endosperm colocalizes with phosphorus (P) (Figure 7A). Localization of P usually mirrors phytate distribution in seeds, indicating Fe might be trapped by phytate in the endosperm before it can reach the embryo (Iwai et al., 2012). In summary, the principal storage organ for Fe in Rosid seeds is the embryo; however, Fe reserves can be restrained on the way to the embryo, most likely due to the immobilization by chelation in the endosperm or in the seed coat.

*Carica papaya* showed the most concentrated Fe hotspots among all the analyzed seeds. Despite the presence of a large endosperm, the Fe signal from Perls staining appeared exclusively in the embryo (Figure 5A), specifically localized in homogeneously distributed organelles similar to plastids (Figure 5B). To the best of our knowledge, plastids have never been reported as representing the main Fe reserves in any seeds. For example, Cvitanich et al. (2010) determined a large number of amyloplasts in beans, which even contain Fe-binding ferritin proteins; but these plastids were devoid of Perls stain. In papaya, we found that Fe accumulating organelles are related to protein storage vacuoles based on the absence of ferritin (Figure 5D) and presence of proteins (Figure 5E). Protein storage vacuole is the only vacuole plant seeds has and contain phytate enriched globules, where Fe reserves reside (Lanquar et al., 2005; Regvar et al., 2011). These globules are separated from each other, but often detected as a single aggregate under microscope (Roschzttardtz et al., 2009; Eroglu et al., 2017) which might be an artifact due to fixation (Herman and Larkins, 1999). Interestingly, in papaya, proteins aggregated in separated compartments (Figure 5). These compartments seemed to localize in the cytosol instead of being enclosed by a protein storage vacuole.

Endodermis represents the major conserved Fe hotspot in distinct plant lineages (Figure 8). In few species, Fe-enriched cells go beyond the single endodermal cell layer to nearby cortex cells [compare Figure 2B with 2D, also see Ibeas et al. (2017)]. Therefore, the question arises whether VIT1 can also localize to cortex cells in addition to the endodermis. VIT1 is able to localize to the inner-most cortex cells in the absence of the endodermis (Roschzttardtz et al., 2009), which may indicate – regardless of whether it is restricted to a single cell layer or not – that the typical ring-shaped Fe localization close proximity to provascular strands is due to VIT1. VIT1 plasticity may not be limited to the different number of cell layers and may extent to different subcellular localization. In beans, instead of the vacuole, Fe accumulated in the cytosol of endodermal cells (Cvitanich et al., 2010), indicating VIT1 orthologs can localize to the plasma membrane (as opposed to tonoplast in Arabidopsis). Taken together, these variations indicate the core signature of VIT1-mediated Fe accumulation in plants is the enrichment around the provasculature, not the confinement in the single cell layer nor the compartmentalization into the vacuole.

Although VIT1-mediated Fe enrichment was conserved, few species lacked this phenotype (Figures 5–8). This may raise the question of whether VIT1 has been lost in these species during the course of evolution. However, this is unlikely due to conservation of a VIT1 domain in species which lack a Fe-enriched endodermis (PF01988) (Figure 4A and Supplementary Figures 2, 3). Alternatively, we hypothesize that other transporters might have taken over VIT1’s function in these species. Studies with loss of function mutants indicate that Fe patterns are eventually determined by a single dominant transporter (Kim et al., 2006; Eroglu et al., 2017). For example, in *A. thaliana*, VIT1’s presence prevents another protein, MTP8, from storing Fe. Likewise, when a more preferential Fe transporter is present, VIT1 may still mediate Fe accumulation (i.e., Fe-enriched endodermis) but to a much lesser extent. Furthermore, the nature of techniques used in the study is biased in revealing a less pronounced Fe store in the presence of a highly pronounced one, making it likely to miss VIT1’s impact in the presence of a stronger metal transporter. For instance, X-ray analysis (Kim et al., 2006) or Perls/DAB staining (Figure 1A) fail to detect any Fe in cortex or epidermal cells in the presence of the large endodermal Fe pool, although they constitute half of the total seed Fe (Ramos et al., 2013). Taken together, VIT1 and its associated phenotype is well-conserved despite some seeds showing alternative Fe hotspots.

Rosids is a huge lineage including 80,000 species belonging to 147 families, comprising more than a third of all angiosperms (Soltis, 2005; Hedges and Kumar, 2009). The current study shows Rosid seeds store Fe in the embryo. This Fe is not equally distributed but most often concentrated in the innermost cell layers, the endodermis and sometimes the cortex, in a VIT1-dependent manner. This phenotype goes beyond dicots and may extend to monocots as shown in rice. Future studies should examine other clades beyond Rosids to pinpoint at which stage of plant evolution the Fe-enriched endodermis appeared as a new feature.

## Author Contributions

SE and NK collected the seeds and performed the histochemistry. KV-M and AK carried out the X-ray analyses. EF performed the bioinformatics. SE and BT conceived and planned the project. SE wrote the manuscript with the contributions of all the authors.

## Conflict of Interest Statement

The authors declare that the research was conducted in the absence of any commercial or financial relationships that could be construed as a potential conflict of interest.

## Footnotes

1 https://phytozome.jgi.doe.gov/pz/portal.html#

2 https://pfam.xfam.org/

3 https://web.expasy.org/protparam/

4 http://www.enzim.hu/hmmtop/

5 https://blast.ncbi.nlm.nih.gov/Blast.cgi?PAGE=Proteins
