# Supplementary material for: The Conservation of VIT1-Dependent Iron Distribution in Seeds
Source: Front Plant Sci. 2019 Jul 12;10:907. doi: 10.3389/fpls.2019.00907 (PMC6640190; doi:10.3389/fpls.2019.00907)
Supplement: Supplementary file 1 [file Table_1.DOCX]

**Supp. Fig. 1.** Alignments of 34 VIT1 sequences from 18 different plant species.


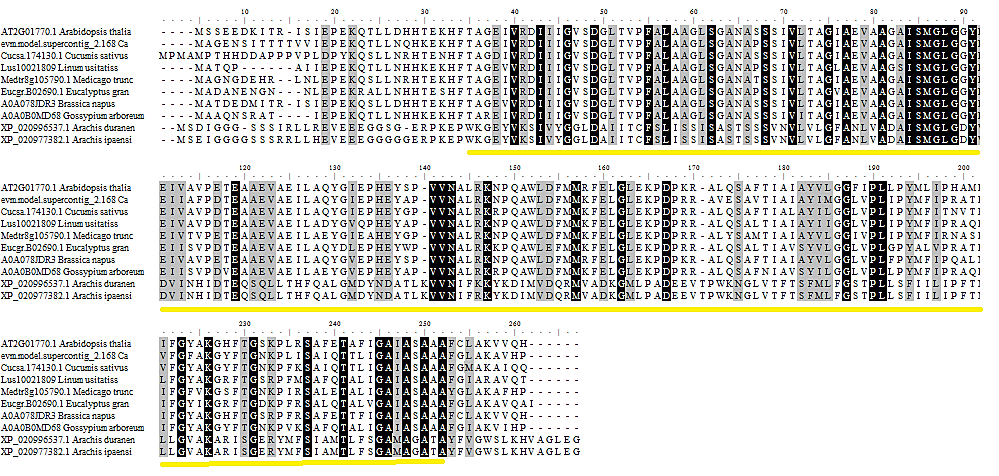


**Supp. Fig. 2.** Alignment of VIT1 from species which were chosen for seed Fe staining. The yellow line shows the conserved VIT1 (PF01988) domain structure

**A**


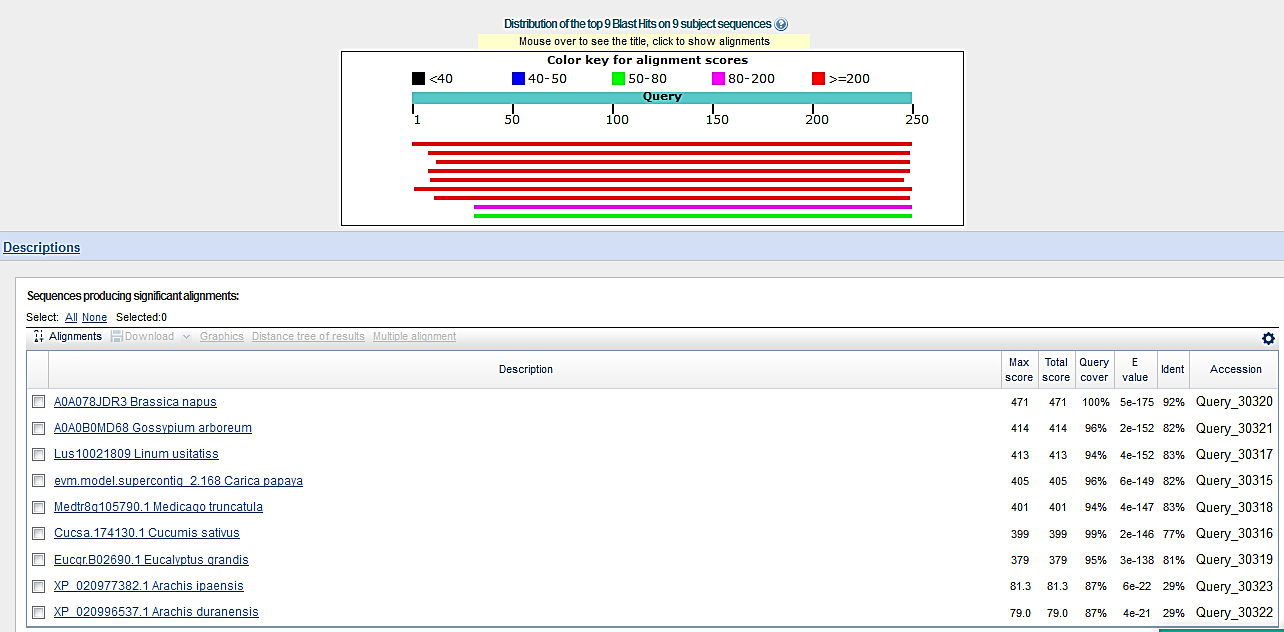


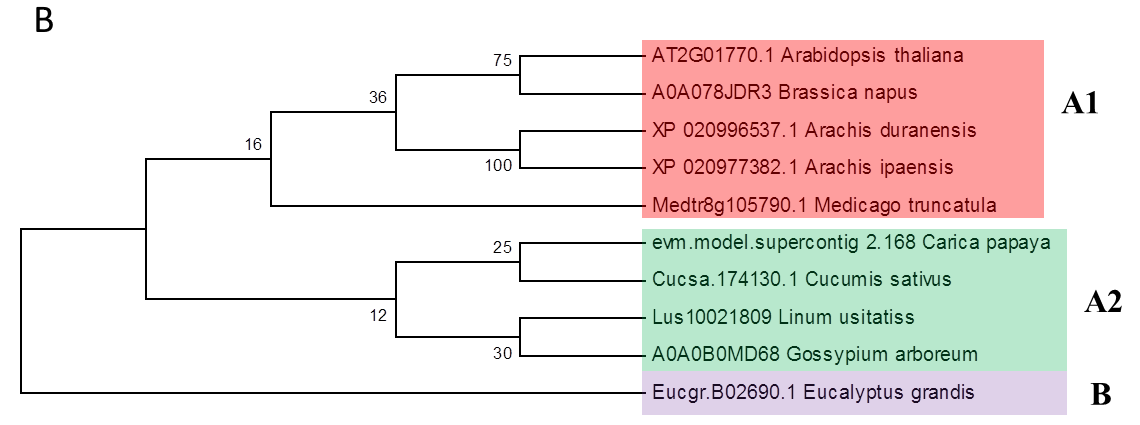


**Supp. Fig. 3.** Comparison of *A. thaliana* with the other nine plant VIT1 sequences using protein blast.

**
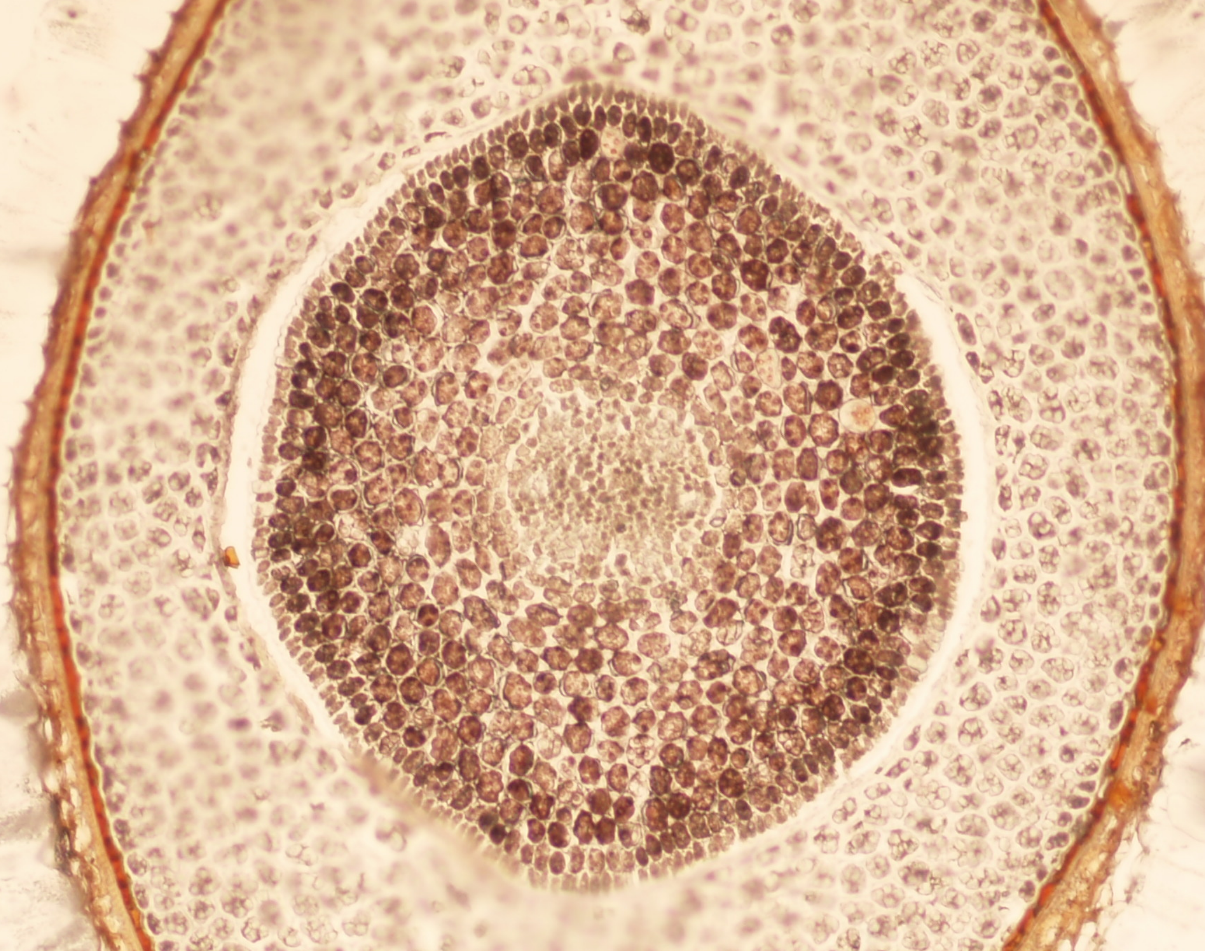
**

**Supp. Fig. 4.** Perls/DAB staining of common flax (*Linum usiatiss*).

**Supp. Table 1.** Biochemical properties of VIT1s according to their protein sequence analyses.
